# Nurses’ Adherence to Patient Safety Principles: A Systematic Review

**DOI:** 10.3390/ijerph17062028

**Published:** 2020-03-19

**Authors:** Mojtaba Vaismoradi, Susanna Tella, Patricia A. Logan, Jayden Khakurel, Flores Vizcaya-Moreno

**Affiliations:** 1 Faculty of Nursing and Health Sciences, Nord University, 8049 Bodø, Norway; 2 Faculty of Health and Social Care, LAB University of Applied Sciences, 53850 Lappeenranta, Finland; susanna.tella@saimia.fi; 3 Faculty of Science, Charles Sturt University, 2795 Bathurst, Australia; plogan@csu.edu.au; 4 Research Centre for Child Psychiatry, Department of Child Psychiatry, Faculty of Medicine, University of Turku, 20014 Turku, Finland; jayden.khakurel@utu.fi; 5 Nursing Department, Faculty of Health Sciences, University of Alicante, 03080 Alicante, Spain; flores.vizcaya@ua.es

**Keywords:** adherence, quality of care, patient-safety principles, nursing intervention, practice errors, safe care

## Abstract

*Background:* Quality-of-care improvement and prevention of practice errors is dependent on nurses’ adherence to the principles of patient safety. *Aims:* This paper aims to provide a systematic review of the international literature, to synthesise knowledge and explore factors that influence nurses’ adherence to patient-safety principles. *Methods:* Electronic databases in English, Norwegian, and Finnish languages were searched, using appropriate keywords to retrieve empirical articles published from 2010–2019. Using the theoretical domains of the Vincent’s framework for analysing risk and safety in clinical practice, we synthesized our findings according to ‘patient’, ‘healthcare provider’, ‘task’, ‘work environment’, and ‘organisation and management’. *Findings:* Six articles were found that focused on adherence to patient-safety principles during clinical nursing interventions. They focused on the management of peripheral venous catheters, surgical hand rubbing instructions, double-checking policies of medicines management, nursing handover between wards, cardiac monitoring and surveillance, and care-associated infection precautions. Patients’ participation, healthcare providers’ knowledge and attitudes, collaboration by nurses, appropriate equipment and electronic systems, education and regular feedback, and standardization of the care process influenced nurses’ adherence to patient-safety principles. *Conclusions:* The revelation of individual and systemic factors has implications for nursing care practice, as both influence adherence to patient-safety principles. More studies using qualitative and quantitative methods are required to enhance our knowledge of measures needed to improve nurse’ adherence to patient-safety principles and their effects on patient-safety outcomes.

## 1. Introduction

The World Health Organization defines patient safety as the absence of preventable harm to patients and prevention of unnecessary harm by healthcare professionals [1]. It has been reported that unsafe care is responsible for the loss of 64 million disability-adjusted life years each year across the globe. Patient harm during the provision of healthcare is recognized as one of the top 10 causes of disability and death in the world [2]. Regarding the financial consequence of patient harm, a retrospective analysis of inpatient harm based on data collected from 24 hospitals in the USA showed that harm-reduction strategies could reduce total healthcare costs by $108 million U.S. and generate a saving of 60,000 inpatient care days [3]. Additionally, the loss of income and productivity due to other associated costs of patient harm are estimated to be trillions of dollars annually [4]. The burden of practice errors on patients, their family members, and the healthcare system can be reduced through implementing patient-safety principles based on preventive and quality-improvement strategies [5]. Patient-safety principles are scientific methods for achieving a reliable healthcare system that minimizes the incidence rate and impact of adverse events and maximizes recovery from such incidents [6]. These principles can be categorized as risk management, infection control, medicines management, safe environment and equipment [7], patient education and participation in own care, prevention of pressure ulcers, nutrition improvement [8], leadership, teamwork, knowledge development through research [9], feeling of responsibility and accountability, and reporting practice errors [10].

The nurses’ role is to preserve patient safety and prevent harm during the provision of care in both short-term and long-term care settings [11,12]. Nurses are expected to adhere to organizational strategies for identifying harms and risks through assessing the patient, planning for care, monitoring and surveillance activities, double-checking, offering assistance, and communicating with other healthcare providers [13,14]. In addition to clear policies, leadership, research driven safety initiatives, training of healthcare staff, and patient participation [1,15], nurses’ adherence to the principles of patient safety [16,17] is required for the success of interventions aimed at the prevention of practice errors and to achieve sustainable and safer healthcare systems.

### Background

Adherence to and compliance with guidelines and recommendations are influenced by personal willingness, culture, economic and social conditions, and levels of knowledge [18,19]. On the other hand, lack of adherence and compliance contravenes professional beliefs, norms, and expectations of the healthcare professional’s role [20].

Institutional systemic factors influencing nurses’ adherence to and compliance with patient-safety principles are as follows: the organizational patient-safety climate [21], workload, time pressure, encouragement by leaders and colleagues [22,23,24], level of ward performance [25], provision of education for the improvement of knowledge and skills [11,18], institutional procedures or protocols, and also communication between healthcare staff and patients [11]. In addition, personal motivation, resistance to change, feelings of autonomy, attitude toward innovation, and empowerment are personal factors that impact on the nurses’ adherence to patient-safety principles [26].

A theoretical framework for analysing risk and safety in healthcare practice has been devised by Vincent et al. (1998) [27] based on the Reason’s model of organizational accidents [28]. It combines ‘person-centred’ approaches, where the focus is on individual responsibility for the preservation of patients’ safety and prevention of their harm, and the ‘system-centred’ approach, which considers organizational factors as precursors for endangering patient safety [29]. According to this theoretical framework, initiatives aimed at the improvement of patient safety require systematic assessments and integrative interventions to target different elements in the hierarchy of the healthcare system, including patient, healthcare provider, task, work environment, and organization and management. This framework, and similar models for risk and safety management, can help with the analysis of patient harm, to identify probable pitfalls, as well as explore how to prevent future similar incidents [30].

Adherence to the principles of patient safety and the prevention and reduction of practice errors have been facilitated by technological solutions in recent years [31,32]; however, suboptimal quality and safety of care remain evident, indicating the need for improved understandings of the various factors and conditions that increase adherence in daily nursing practice [33]. Consequently, this review aimed to retrieve, explore, and synthesise factors evident in the international literature that influenced nurses’ adherence to patient-safety principles. Vincent’s framework was used for the classification of findings, in order to systematically present the findings and inform clinical practice.

## 2. Materials and Methods

### 2.1. Design

A systematic review was conducted. It is an explicit and clear method of data collection, systematic description, and synthesis of findings, to reach the study goal [34,35,36]. The review findings are presented narratively since heterogeneities in the methods, objectives, and results of studies that met the inclusion criteria did not lend themselves to meta-analysis. The Preferred Reporting Items Systematic Reviews and Meta-analysis (PRISMA) Statement (2009) was applied to inform this systematic review [36].

### 2.2. Search Methods

Search keywords were determined after team discussions, performing a pilot search in general and specialized databases, and consultation with a librarian. Key search terms relating to adherence to patient-safety principles by nurses were used to conduct a Boolean search. For operationalising the study concept, the definition of adherence as a behaviour carried out actively by people according to orders or advice was used [37]. The word adherence is used interchangeably with, and sometimes at the same time as, the word compliance, since both can indicate the outcome of care interactions between the healthcare provider and the caregiver [38,39,40,41,42]. However, adherence indicates responsibility and empowerment on the healthcare professional’s part to actively perform the expected behaviour compared to compliance that shows responsibility on the patient’s part to follow up the therapeutic regimen [43,44].

The search was limited to the time period of January 2010 to August 2019, in English scientific journals available through the following online databases: PubMed (including Medline), CINAHL, Scopus, Web of Science, PsycINFO, ProQuest, and EBSCO. In addition, the authors performed searches in Nordic and Finnish databases to improve the search coverage. To find relevant studies for inclusion in the data analysis and synthesis, inclusion criteria for selection were articles with a focus on adherence to patient-safety principles in clinical nursing interventions published in online peer-reviewed scientific journals. Articles on patients and other healthcare providers, or on non-clinical initiatives, or that had no exact relevance to adherence to patient-safety principles were excluded.

### 2.3. Search Outcome and Data Extraction

The authors (M.V., S.T., J.K., and F.V.M.) independently performed each step of the systematic review, holding frequent online discussions and making collective agreements on how to proceed through the review steps. Gray literature, such as unpublished dissertations and policy documents and cross-referencing from bibliographies, were assessed, to improve the search coverage. Guidance and support with the search process were obtained from the librarian, when needed. All authors independently screened the titles, abstracts, and full texts of the studies retrieved during the search process. In the cases where disagreements about the inclusion of selected studies occurred, discussions were held until a consensus was reached.

A data extraction table was used to collect data on the characteristics of studies. The table included the lead author’s name, publication year, country, design, sample size and setting, and information relating to adherence to patient-safety principles. Prior to the full data extraction, this table was pilot-tested with a few selected studies, to ensure that data relevant to the review aim and analysis would be appropriately gathered.

### 2.4. Quality Appraisal

The selected articles were appraised based on the appropriateness of the research structure using the evaluation tools provided by the Enhancing the QUAlity and Transparency of health Research (EQUATOR) website [45] and criteria outlined by Hawker et al. (2002) [46], addressing the study aim, research structure, theoretical/conceptual research framework, conclusion, and references. The appraisal tool appropriate to cross-sectional, observational and cohort studies such as the Strengthening the Reporting of Observational Studies in Epidemiology (STROBE) was used to evaluate the suitability of selected studies for inclusion in the final data synthesis and analysis. The researchers believed that the quality appraisal items for determining the inclusion of a study in the final dataset did not align to a scoring system; therefore, they used a yes/no system to answer the appraisal-tool items during the quality appraisal and held frequent discussions on the importance and quality of each article before making the final decision on the selection of studies for data analysis and synthesis.

### 2.5. Data Abstraction and Synthesis

The Vincent’s framework for analysing risk and safety in clinical practice [27,47] was used to organize and connect the review findings to the wider theoretical perspective of patient safety. This framework was developed based on the Reason’s organisational accident model [28]. Accordingly, issues in patient safety originate in various systemic features at different categories of patient, healthcare provider, task, work environment, and organisation and management [27,47]. The use of this framework helped with the description and categorisation of data retrieved and accommodated heterogeneities in the studies retrieved, with respect to method, samples, settings, and findings, facilitating the integrative presentation of the review findings. The authors (M.V., S.T., P.A.L., J.K., and F.V.M.) reviewed the included studies, to allocate the studies’ findings to each category, and used frequent discussions to reach a consensus.

## 3. Results

### 3.1. Search Results and Study Selections

**Figure 1:**
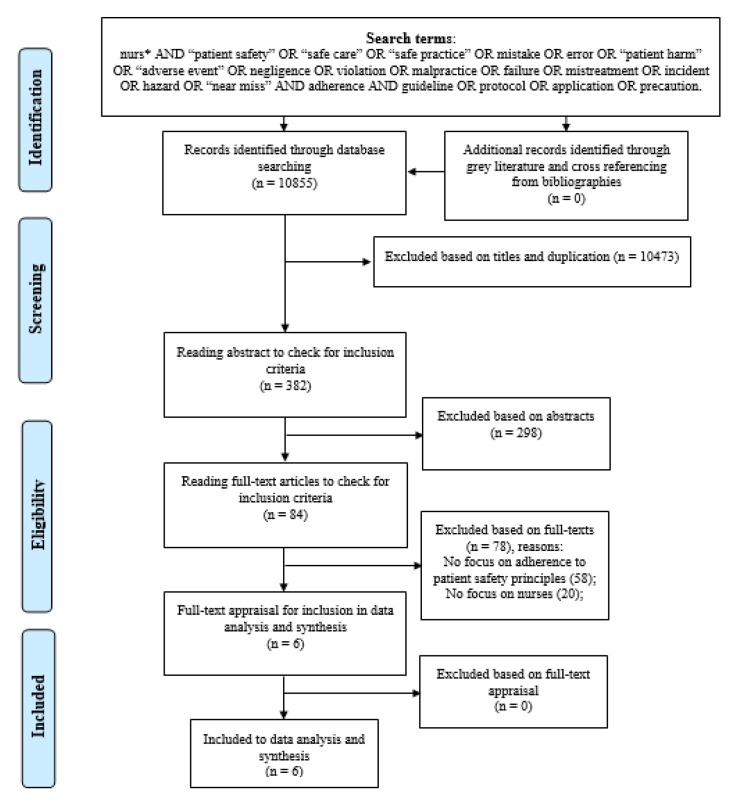
The study flow diagram according to the Preferred Reporting Items for Systematic Reviews and Meta-Analyses (PRISMA).

The thorough literature search using the key terms led to the retrieval of 10,855 articles. After deleting irrelevant and duplicate titles, 382 entered the abstract-reading phase. Each abstract was assessed by using the inclusion criteria, resulting in 84 possibly relevant articles. The full texts were obtained from Finnish and Norwegian libraries and were carefully read to select only those articles that had a precise focus on adherence to patient-safety principles during clinical nursing interventions by nurses. This resulted in the final six articles chosen for data analysis. Excluded studies were on adherence by other healthcare providers, rather than nurses, or had no exact relevance to patient-safety principles. The methodological quality of the selected articles was assessed during the full-text appraisal, and no article was excluded. In general, they had acceptable qualities with respect to study research structure, theoretical and conceptual research frameworks, and relevant findings to the review aim. Grey literature and the manual search in the reference lists of the selected studies led to no more articles being discovered for inclusion. Appendix A presents the search results, giving the number of articles located in each database. The Preferred Reporting Items for Systematic Reviews and Meta-Analyses (PRISMA) flowchart is shown in Figure 1.

### 3.2. General Characteristics of the Selected Studies

The general characteristics of the selected studies (*n* = 6) are presented in Table 1. The studies were published from 2014 to 2019 and were conducted in Australia [48], Finland [49], Norway [50], South Korea [51], Sweden [52], and the UK [53].

Three studies used a survey design [50,51,52]; one study used an observational method [53]; one applied an observational intervention design [49]; and another one was a three-stage pre-post time-series study [48]. Except for one study [49] that was published in the Finnish language, all other articles were written in English.

Diverse foci were evident in the studies: adherence to patient-safety principles on the management of peripheral venous catheters [52], surgical hand rubbing instructions [49], double-checking policies of medicines’ preparation and administration [53], handover from the intensive care unit (ICU) to the cardiac ward [48], cardiac monitoring and surveillance standards [50], and care-associated infection precautions [51].

### 3.3. Findings of Studies with Connection to the Vincent’s Framework

**Figure 2:**
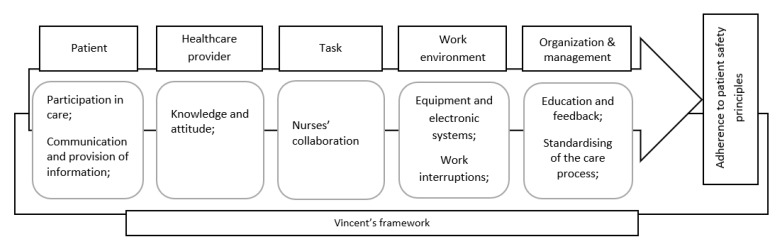
Schematic model of nurses’ adherence to patient-safety principles based on the Vincent’s framework.

The findings were classified based on the theoretical framework for analysing risk and safety in clinical practice developed by Vincent (1998, 2010) [27,47] and grouped by factors related to the patient, healthcare provider, task, work environment, and organisation and management. Variations in the findings within the selected studies related to the type of patient-safety principles or different clinical settings facilitated the description and synthesis of findings under the above-mentioned categories (Figure 2).

#### 3.3.1. Patient

This category was about the role of patients and how they could impact nurses’ adherence to patient-safety principles. For instance, errors made during medicines’ preparation and administration, and a deviation from medication safety principles by nurses were reported. The deviation with a high possibility of endangering patient safety happened where the parents of patients or their companions were left unobserved and unsupervised by nurses to administer medicines to patients. Unobserved or unsupervised administration contravenes the medicines management principle, which requires a nurse’s direct supervision; a crucial consideration for the prevention of abuse and patient avoidance of taking medicines as prescribed [53]. Moreover, in spite of the emphasis on patient participation in patient-safety activities, nursing handovers were delivered mainly outside the patient’s room [48], or no information was provided to patients regarding the purpose and process of cardiac monitoring [50]. These deviations could hinder patients’ active involvement in their own safe care. Additionally, the only communication line between patients and nurses was the call bell, and nurses rarely questioned patients about their pain or comfort. These identified issues represent missed opportunities for the nurses’ continuous observation role for early detection and prevention of harm during handovers from the ICU to the cardiac ward [48].

#### 3.3.2. Healthcare Provider

This category described how nurses’ knowledge and attitudes were associated with their adherence to patient-safety principles. Variations in nurses’ adherence to patient-safety principles could be attributed to their varied levels of knowledge and attitudes. Examples included nurses’ incomplete adherence to infection-control principles, which encompassed the daily inspection of peripheral venous catheter sites, surgical hand rubbing, disinfection of hands, and the use of disposable gloves and aprons when exposed to patient excretions [49,51,52]. Other examples were related to the principles of medicines’ management: inappropriate speed of intravenous bolus, incorrect medicines’ preparation, administration at incorrect times, problematic labelling of flush syringes and administration of intravenous antibiotics without flushing, not receiving the medicines’ complete dose by patients, and incorrect mixing of medicines with diluent [53]. Lack of sufficient knowledge and skills regarding cardiac monitoring and surveillance standards were also evident, with incorrect placement of cardiac electrodes and/or skin preparation before the procedure leading to inconsistent monitoring, which could endanger patient safety [50]. Interestingly, being a newly graduated nurse with less time having passed since obtaining the nursing certificate was associated with better adherence to the peripheral venous catheter-care principles, possibly due to having more informatics skills and updated knowledge of nursing care and following up of rules set by senior nurses [52]. Additionally, negative attitudes and perceptions toward the significance of care standards, individual aesthetic manicure preferences, and the presence of eczema and skin wounds hindered adherence to the surgical hand rubbing protocol, thus having negative implications for patient safety [49].

#### 3.3.3. Task

In this category, the association between the identity and type of nursing task and adherence to patient-safety principles by nurses was considered. The lowest adherence rates were evident in ‘independent’ medicine management tasks such as dose calculation, rate of administering intravenous bolus drugs, and labelling of flush syringes. On the other hand, a higher rate of adherence was reported for ‘cooperative’ tasks with higher levels of complexity, such as the double-checking of drugs for the actual administration of medicine to the patient [53]. Similarly, a higher number of nurses working and collaborating together in the ward was associated with a higher rate of adherence to infection-control precautions, including putting sharp articles into appropriate boxes, covering both the mouth and nose, and disinfection of hands after glove removal [51].

#### 3.3.4. Work Environment

The effect of equipment and the workplace condition on adherence to patient-safety principles was reported in this category. The availability of equipment and electronic resources and digitalization increased the likelihood of adherence to patient safety principles related to medicine management [53], peripheral venous catheter care [52], and cardiac monitoring and surveillance [50]. Accordingly, a telemetry cover on cardiac telemetry and monitoring units helped with the prevention of nosocomial infection by preventing contamination of shared equipment [50]. Electronic resources and digitalization helped with reminding the daily inspection and information-sharing between nurses regarding peripheral venous catheter insertion sites [52]. The existence of an environmental space for preparation of medicines without interruptions helped nurses adhere more closely to double-checking instructions of preparation and administration on weekends, as compared with weekdays [53].

#### 3.3.5. Organisation and Management

This category focused on collaboration between nurses and the leadership role in motivating nurses’ adherence to patient-safety principles. As an example, adherence to the surgical hand rubbing principles, including properly drying hands after alcohol hand rubbing and washing with water and soap, and alcohol hand rubbing up to elbows, was improved after the provision of feedback by nurse leaders [49]. Regular practical feedback processes, interaction opportunities and observation of peers and senior colleagues, and leadership motivated nurses’ adherence to daily inspection of the peripheral venous catheter site and the use of disposable gloves when handling peripheral venous catheters insertion sites [52]. Adherence to patient-safety principles by cardiac nurses was improved through feedback provision and informing nurses in the ICU of the type of nursing interventions conducted in cases of serious dysrhythmias and their outcomes [50].

The provision of a standard process for handover, such as the introduction of a validated handover tool, improved nurses’ readiness to receive patients from the ICU. It informed the preparation of the required equipment for care, enabled performance of handovers at the patient bedside, and involved patients in their care, while also assisting with attending patients’ needs, checking patients’ identity, and collecting data of their medical history and allergies. Further, the standardising of the handover process helped with the continuity of care plan by formalising discussions between nurses and assisting with removal of any ambiguities, so increasing awareness of risks to patient safety [48]. The higher adherence rate to standard precautions for infection control were found when there was a higher nurse-to-patient ratio indicating the association between workload and patient-safety management [51]. Similarly, the development of a local practice standard for cardiac monitoring and surveillance, as well as for assessing the eligibility of patients for admission to critical and non-critical telemetry sections, would improve adherence to patient-safety principles for the cardiac patient [50].

## 4. Discussion

This systematic review integrated current international knowledge through the categorization of factors affecting adherence to patient-safety principles by nurses to the elements of the Vincent’s framework (1998 and 2010) for analysing risk and safety in clinical practice [27,47].

In this review, leaving patients’ companions unsupervised during medicines’ administration, performing handovers outside patients’ rooms, and lack of the provision of information and appropriate communication with patients hindered patient participation in their understandings of their own care. Lack of engagement of patients in safe-care initiatives contravenes nurses’ adherence to patient-safety principles. Benefiting from patients’ participation requires understanding of how to improve the patient’s willingness to act as an active member of the healthcare team, development of practical guidelines for such an engagement with the consideration of patients and their relatives’ knowledge and skills of the care process, as well as definition of the role and provision of supervision and guidance by nurses. The assigned participation task should be communicated appropriately to the patient, have congruity with patients’ knowledge of nursing routines and their own implementation capacity, as well as be incorporated into routine care with the consideration of infrastructures and healthcare missions [14,54,55]. It has been suggested that planning and performing nursing care at the patient’s bedside can improve patient participation, reduce work interruptions [56], and consequently improve nurses’ adherence to safe care guidelines [11].

The findings of this review highlighted that nurses’ knowledge, perceptions, and attitudes influenced their adherence to patient-safety principles. Nurses have multiple roles and central responsibility to keep patients safe in the complex healthcare environment [57,58]. The effect of personal and professional values and attitudes on the consistency of adherence to patient safety by nurses has been shown to be more important than the effect of their workloads [22]. It is believed that individual factors such as nurses’ attitudes, perceptions, knowledge, and information seeking can facilitate or hinder the use of clinical practice guidelines by nurses and consequently endanger patient safety [11,26] through inconsistent adherence to patient-safety principles [59].

It was evident that collaborative tasks fostered nurses’ adherence to patient-safety principles. Improving nurses’ knowledge of tasks improves nurses’ adherence [60]. Moreover, the coordinated management approach and collaboration with team members enhance the effectiveness of patient-safety interventions due to the creation of a shared understanding of changes that should be made by all healthcare staff to improve the quality of care [61,62].

With regard to the work environment, the findings of this review highlighted how equipment and electronic systems could assist with sharing information between healthcare providers and enhance adherence to patient-safety principles. One part of the healthcare system’s commitment to patient safety is the preparation of appropriate work equipment [63,64]. Technology can support data security and facilitate nursing care through the provision of real-time and ubiquitous documentation, which is needed for professional interactions and collaboration [65]. Digital systems can reduce the time needed to perform nursing care and limit errors in drug administration, as well as improve nurses’ and patients’ satisfaction with care [66,67].

An appropriate work environment was characterised as one where nurses were less interrupted, and lower workloads improved adherence to patient-safety principles. An appropriate work environment is associated with better patient safety and less burnout. Workload and burnout act as negative mediators of safe care [68,69]. A work environment characterised by a heavy workload and mental pressure [23,24,70] and frequent disruptions [71] has been implicated in reducing nurses’ adherence to safety-related principles. There is an association between patient safety and the nurses’ work environment [39,72,73] and implementation of patient-safety principles to prevent errors and adverse events [26,74].

The findings of this review emphasized the role of regular education and provision of feedback to nurses. Taking responsibility for actions and behaviours through education and feedback is a crucial aspect of professional practice [75]. The empowerment of nurses to intervene based on care standards is an expectation of healthcare leaders which can be achieved through the development of the culture of patient safety [33,76,77,78], the implementation of educational programs, and timely feedback and reminders [79,80,81]. Further, the use of standard processes, supported by validated tools, guided nurses and facilitated their adherence to patient-safety principles. Usability, format, easy access of the contents of guidelines, and consideration of time, staffing, chain of communication, accuracy of practice, supplies of equipment, and logistics are the main advantages of guidelines that facilitate the implementation of safe care [26,82].

### Limitations and Suggestions for Future Studies

In spite of the emphasis on adherence to patient-safety principles and patient-care outcomes, this study has directly focused on nurses’ adherence to patient-safety principles, which can impact our understandings of the variation of factors influencing this important concept. However, the wide nature of the search in the electronic databases and in various languages convinced the researchers that the study topic has been addressed appropriately and an answer based on the current knowledge can be provided. However, the limited number of studies that met the inclusion criteria for this review hinders the full exploration of the relationship between individual and systemic factors that impact on nurses’ adherence to patient-safety principles in inpatient and outpatient settings.

## 5. Conclusions

This review has shown that adherence to patient-safety principles was affected by numerous intersecting and complex factors. Variations in the studies’ aims, methods, and results hinder the formation of a determinant conclusion on how adherence to patient-safety principles can be improved. However, based on the review results, general indications are that improvement of nurses’ knowledge about patient safety, collaboration in performing tasks, reduction of workloads, provision of appropriate equipment and electronic systems for communication and sharing information, regular feedback in the workplace, and standardization of the care processes can help with enhancing nurses’ adherence to patient-safety principles. Future qualitative and quantitative studies are needed to better understand how to promote and mitigate adherence to safe-care principles by clinical nurses.

## Figures and Tables

**Table 1:** Characteristics of selected studies for data analysis and synthesis.

Authors, Year, Country	Aim	Method	Sample and Setting	Main Finding	Conclusion
Förberg et al., 2014, Sweden [52]	To investigate nurses’ adherence to the clinical practice guidelines regarding peripheral venous catheters and investigate their understandings of work context influencing it.	Survey	A children’s hospital with 245 beds, 373 nurses from 23 medical and surgical inpatient, intensive care, the operating, anaesthetic, advanced homecare, and outpatient wards.	The importance of the workplace condition in terms of information sharing and feedback.	The need for various strategies for improving adherence among nurses.
Rintala et al., 2014, Finland [49]	To evaluate adherence to surgical hand rubbing directives among operating room personnel, in public hospitals in Southwest Finland.	Observational before-after intervention	11 surgical settings of four hospitals, 190 and 73 nurses in the first and second observation rounds, respectively.	The relative impact of the feedback intervention on adherence by nurses.	Necessity of effective educational methods and role models.
Alsulami et al., 2014, UK [53]	To explore the follow-up of double-checking policies by nurses and assess the identity of medication-administration errors despite double-checking.	Prospective observational	Medical and surgical wards, the PICU and NICU, observation of preparation and administration of 2000 drug doses to 876 children.	Deviations from the policies of medication administration.	Encouragement of double-checking steps during medication administration, and prevention of interruptions.
Graan et al., 2016, Australia [48]	To investigate the adoption of standardised nursing handover guidelines from the ICU to the cardiac ward in regard to understanding risks to patient safety before and after the implementation.	Three-stage, pre–post time series, and focus group interviews pre-and/or post-implementation.	A metropolitan private hospital with a 15-bed ICU and a 46-bed cardiac surgical ward; 20 consecutive episodes of ICU-to-ward handover and a further 20 post-implementation episodes; A purposive sample of 19 senior nurse managers and clinicians.	Unsafe practice of handover interventions and information gap.	The need for the adoption of standardised handover tools for reducing handover variabilities.
Fålun et al., 2019, Norway [50]	To study cardiovascular nurses’ knowledge of, and adherence to, practice standards for cardiac surveillance and their knowledge improvements over time, in years 2011 and 2017.	Survey	363 nurses from 44 hospitals in 2011 and 38 hospitals in 2017.	Failure to fully adhere to cardiac telemetry monitoring standards.	Developing educational programmes regarding the safe practice of cardiac monitoring.
Lim et al., 2019, South Korea [51]	To investigate nurses’ adherence to standard precautions and its association with their perceptions of safe care.	Cross-sectional	329 nurses working in a teaching hospital.	Intermediate adherence to standard precautions.	Devising integrative curricula to improve nurses’ transition to professional practice.

PICU: paediatric intensive care unit; NICU: neonatal intensive care unit; ICU: intensive care unit.

## Appendix A

**Table A1:** Search strategy and results based on each database.

Database	Total in Each Database	Selection Based on Title Reading	Selection Based on Abstract Reading	Selection Based on Full-Text Reading/Appraisal
ProQuest	3169	0	0	0
CINAHL	4271	40	8	1
EBSCO	673	7	5	0
PubMed [including Medline]	33	27	20	1
PsycINFO	442	42	6	0
Scopus	1387	203	33	2
Web of Science	856	62	11	1
*Norwegian databases*				
Oria	4	0	0	0
Idunn	0	0	0	0
Norart	0	0	0	0
Helsebiblioteket.no	1	0	0	0
Cristin	4	0		
Finnish database—Medic	15	1	1	1
Manual search/backtracking references	0	0	0	0
**Total of databases**	10855	382	84	6

## References

[1] World Heath Organization (WHO) (2019). Patient Safety. https://www.who.int/patientsafety/en/.

[2] Harvard Global Health Institute Patient Safety: A Major Public Health Challenge. https://globalhealth.harvard.edu/qualitypowerpoint.

[3] Adler L., Yi D., Li M., McBroom B., Hauck L., Sammer C., Jones C., Shaw T., Classen D. (2018). Impact of Inpatient Harms on Hospital Finances and Patient Clinical Outcomes. J. Patient Saf..

[4] Slawomirski L., Auraaen A., Klazinga N.S. The Economics of Patient Safety. https://www.oecd-ilibrary.org/social-issues-migration-health/the-economics-of-patient-safety_5a9858cd-en.

[5] Rodziewicz T.L., Hipskind J.E. (2020). Medical Error Prevention. StatPearls.

[6] Emanuel L., Berwick D., Conway J., Combes J., Hatlie M., Leape L., Reason P., Schyve P., Vincent C., Walton M. (2008). What Exactly is Patient Safety. Advances in Patient Safety: New Directions and Alternative Approaches.

[7] Sibal A., Uberoi R.S., Malani A. (2016). An approach to improve patient safety and quality beyond accreditation. World Hosp. Health Serv..

[8] Mitchell P. (2008). Defining Patient Safety and Quality Care. Patient Safety and Quality: An Evidence-Based Handbook for Nurses.

[9] Kanerva A., Kivinen T., Lammintakanen J. (2017). Collaborating with nurse leaders to develop patient safety practices. Leadersh Health Serv..

[10] Dixon-Woods M. (2010). Why is Patient Safety so Hard? A Selective Review of Ethnographic Studies. J. Health Serv. Res. Policy.

[11] Lin F., Gillespie B.M., Chaboyer W., Li Y., Whitelock K., Morley N., Morrissey S., O’Callaghan F., Marshall A.P. (2019). Preventing surgical site infections: Facilitators and barriers to nurses’ adherence to clinical practice guidelines—A qualitative study. J. Clin. Nurs..

[12] Sermeus W. (2016). Understanding the role of nurses in patient safety: From evidence to policy with RN4CAST. BMC Nurs..

[13] Henneman E.A. (2017). Recognizing the Ordinary as Extraordinary: Insight into the “Way We Work” to Improve Patient Safety Outcomes. Am. J. Crit. Care.

[14] Vaismoradi M., Jordan S., Kangasniemi M. (2015). Patient participation in patient safety and nursing input—a systematic review. J. Clin. Nurs..

[15] International Council of Nurses (ICN) (2019). Patient Safety-ICN Position. https://www.icn.ch/sites/default/files/inline-files/D05_Patient_Safety_0.pdf.

[16] Rashvand F., Ebadi A., Vaismoradi M., Salsali M., Yekaninejad M.S., Griffiths P., Sieloff C. (2017). The assessment of safe nursing care: Development and psychometric evaluation. J. Nurs. Manag..

[17] Vaismoradi M., Salsali M., Turunen H., Bondas T. (2013). A qualitative study on Iranian nurses’ experiences and perspectives on how to provide safe care in clinical practice. J. Res. Nurs..

[18] Efstathiou G., Papastavrou E., Raftopoulos V., Merkouris A. (2011). Factors influencing nurses’ compliance with Standard Precautions in order to avoid occupational exposure to microorganisms: A focus group study. BMC Nurs..

[19] Haynes R.B., Sackett D.L., Taylor D.W. (1979). Compliance in Health Care.

[20] Playle J.F., Keeley P. (1998). Non-compliance and professional power. J. Adv. Nurs..

[21] Hessels A.J., Larson E.L. (2016). Relationship between patient safety climate and standard precaution adherence: A systematic review of the literature. J. Hosp. Infect..

[22] Jam R., Mesquida J., Hernández Ó., Sandalinas I., Turégano C., Carrillo E., Pedragosa R., Valls J., Parera A., Ateca B. (2018). Nursing workload and compliance with non-pharmacological measures to prevent ventilator-associated pneumonia: A multicentre study. Nurs. Crit. Care.

[23] Jimmieson N.L., Tucker M.K., White K.M., Liao J., Campbell M., Brain D., Page K., Barnett A.G., Graves N. (2016). The role of time pressure and different psychological safety climate referents in the prediction of nurses’ hand hygiene compliance. Saf. Sci..

[24] Zhang S., Kong X., Lamb K.V., Wu Y. (2019). High nursing workload is a main associated factor of poor hand hygiene adherence in Beijing, China: An observational study. Int. J. Nurs. Pract..

[25] Schutijser B., Klopotowska J.E., Jongerden I., Spreeuwenberg P., Wagner C., de Bruijne M. (2018). Nurse compliance with a protocol for safe injectable medication administration: Comparison of two multicentre observational studies. BMJ Open.

[26] Jun J., Kovner C.T., Stimpfel A.W. (2016). Barriers and facilitators of nurses’ use of clinical practice guidelines: An integrative review. Int. J. Nurs. Stud..

[27] Vincent C., Taylor-Adams S., Stanhope N. (1998). Framework for analysing risk and safety in clinical medicine. BMJ.

[28] Reason J. (2000). Human error: Models and management. BMJ.

[29] van Beuzekom M., Boer F., Akerboom S., Hudson P. (2010). Patient safety: Latent risk factors. BJA Br. J. Anaesth..

[30] Vincent C., Burnett S., Carthey J. (2014). Safety measurement and monitoring in healthcare: A framework to guide clinical teams and healthcare organisations in maintaining safety. BMJ Qual. Saf..

[31] Kutney-Lee A., Kelly D. (2011). The effect of hospital electronic health record adoption on nurse-assessed quality of care and patient safety. J. Nurs. Adm..

[32] Strudwick G., Reisdorfer E., Warnock C., Kalia K., Sulkers H., Clark C., Booth R. (2018). Factors Associated With Barcode Medication Administration Technology That Contribute to Patient Safety: An Integrative Review. J. Nurs. Care Qual..

[33] Hessels A.J., Wurmser T. (2019). Relationship among safety culture, nursing care, and Standard Precautions adherence. Am. J. Infect. Control.

[34] Evans D. (2001). Systematic reviews of nursing research. Intensive Crit. Care Nurs..

[35] Higgins J.P.T., Green S.E. (2011). Cochrane Handbook for Systematic Reviews of Interventions Version 5.1 [updated March 2011]. http://handbook-5-1.cochrane.org/.

[36] Liberati A., Altman D.G., Tetzlaff J., Mulrow C., Gøtzsche P.C., Ioannidis J.P.A., Clarke M., Devereaux P.J., Kleijnen J., Moher D. (2009). The PRISMA statement for reporting systematic reviews and meta-analyses of studies that evaluate healthcare interventions: Explanation and elaboration. BMJ.

[37] Gardner C.L. (2015). Adherence: A concept analysis. Int. J. Nurs. Knowl..

[38] Brown M.T., Bussell J., Dutta S., Davis K., Strong S., Mathew S. (2016). Medication Adherence: Truth and Consequences. Am. J. Med. Sci..

[39] Kim J.M., Suarez-Cuervo C., Berger Z., Lee J., Gayleard J., Rosenberg C., Nagy N., Weeks K., Dy S. (2018). Evaluation of Patient and Family Engagement Strategies to Improve Medication Safety. Patient.

[40] Lam W.Y., Fresco P. (2015). Medication Adherence Measures: An Overview. Biomed. Res. Int..

[41] Roter D.L., Wolff J., Wu A., Hannawa A.F. (2017). Patient and family empowerment as agents of ambulatory care safety and quality. BMJ Qual. Saf..

[42] Sharma A.E., Rivadeneira N.A., Barr-Walker J., Stern R.J., Johnson A.K., Sarkar U. (2018). Patient Engagement in Health Care Safety: An Overview Of Mixed-Quality Evidence. Health Aff..

[43] Bissonnette J.M. (2008). Adherence: A concept analysis. J. Adv. Nurs..

[44] Kyngäs H., Duffy M.E., Kroll T. (2000). Conceptual analysis of compliance. J. Clin. Nurs..

[45] EQUATOR Network (2019). Enhancing the QUAlity and Transparency of health Research. http://www.equator-network.org/.

[46] Hawker S., Payne S., Kerr C., Hardey M., Powell J. (2002). Appraising the evidence: Reviewing disparate data systematically. Qual. Health Res..

[47] Vincent C. (2010). Patient Safety.

[48] Graan S.M., Botti M., Wood B., Redley B. (2016). Nursing handover from ICU to cardiac ward: Standardised tools to reduce safety risks. Aust. Crit. Care.

[49] Rintala E., Laurikainen E., Kaarto A.-M., Routamaa M. (2014). Adherence to surgical hand rubbing directives in a hospital district of Southwest Finland. Suomen Lääkärilehti.

[50] Fålun N., Oterhals K., Pettersen T., Brørs G., Olsen S.S., Norekvål T.M., TELMON-NOR Investigators (2020). Cardiovascular nurses’ adherence to practice standards in in-hospital telemetry monitoring. Nurs. Crit. Care.

[51] Lim J.-H., Ahn J.-W., Son Y.-J. (2019). Association between Hospital Nurses’ Perception of Patient Safety Management and Standard Precaution Adherence: A Cross-Sectional Study. Int. J. Environ. Res. Public Health.

[52] Förberg U., Wallin L., Johansson E., Ygge B.-M., Backheden M., Ehrenberg A. (2014). Relationship between work context and adherence to a clinical practice guideline for peripheral venous catheters among registered nurses in pediatric care. Worldviews Evid. Based Nurs..

[53] Alsulami Z., Choonara I., Conroy S. (2014). Paediatric nurses’ adherence to the double-checking process during medication administration in a children’s hospital: An observational study. J. Adv. Nurs..

[54] Ijkema R., Langelaan M., van de Steeg L., Wagner C. (2014). Do patient characteristics influence nursing adherence to a guideline for preventing delirium?. J. Nurs. Scholarsh..

[55] Tobiano G., Bucknall T., Marshall A., Guinane J., Chaboyer W. (2015). Nurses’ views of patient participation in nursing care. J. Adv. Nurs..

[56] Malfait S., Eeckloo K., Van Biesen W., Van Hecke A. (2019). The effectiveness of bedside handovers: A multilevel, longitudinal study of effects on nurses and patients. J. Adv. Nurs..

[57] Cathro H. (2016). Navigating Through Chaos: Charge Nurses and Patient Safety. J. Nurs. Adm..

[58] Gaffney T.A., Hatcher B.J., Milligan R. (2016). Nurses’ role in medical error recovery: An integrative review. J. Clin. Nurs..

[59] Ribeiro L., Fernandes G.C., Souza E.G.d., Souto L.C., Santos A.S.P.D., Bastos R.R. (2019). Safe surgery checklist: Filling adherence, inconsistencies, and challenges. Rev. Col. Bras. Cir..

[60] Simons P.A.M., Houben R., Benders J., Pijls-Johannesma M., Vandijck D., Marneffe W., Backes H., Groothuis S. (2014). Does compliance to patient safety tasks improve and sustain when radiotherapy treatment processes are standardized?. Eur. J. Oncol. Nurs..

[61] Manser T. (2009). Teamwork and patient safety in dynamic domains of healthcare: A review of the literature. Acta Anaesthesiol. Scand..

[62] O’Brien B., Graham M.M., Kelly S.M. (2017). Exploring nurses’ use of the WHO safety checklist in the perioperative setting. J. Nurs. Manag..

[63] Massey D., Chaboyer W., Anderson V. (2016). What factors influence ward nurses’ recognition of and response to patient deterioration? An integrative review of the literature. Nurs. Open.

[64] Ross C., Rogers C., King C. (2019). Safety culture and an invisible nursing workload. Collegian.

[65] Tunlind A., Granström J., Engström Å. (2015). Nursing care in a high-technological environment: Experiences of critical care nurses. Intensive Crit. Care Nurs..

[66] Lee T.-Y., Sun G.-T., Kou L.-T., Yeh M.-L. (2017). The use of information technology to enhance patient safety and nursing efficiency. Technol. Health Care.

[67] Pirinen H., Kauhanen L., Danielsson-Ojala R., Lilius J., Tuominen I., Díaz Rodríguez N., Salanterä S. (2015). Registered Nurses’ Experiences with the Medication Administration Process. Adv. Nurs..

[68] Liu X., Zheng J., Liu K., Baggs J.G., Liu J., Wu Y., You L. (2018). Hospital nursing organizational factors, nursing care left undone, and nurse burnout as predictors of patient safety: A structural equation modeling analysis. Int. J. Nurs. Stud..

[69] Usher K., Woods C., Parmenter G., Hutchinson M., Mannix J., Power T., Chaboyer W., Latimer S., Mills J., Siegloff L. (2017). Self-reported confidence in patient safety knowledge among Australian undergraduate nursing students: A multi-site cross-sectional survey study. Int. J. Nurs. Stud..

[70] Hall L.H., Johnson J., Watt I., Tsipa A., O’Connor D.B. (2016). Healthcare Staff Wellbeing, Burnout, and Patient Safety: A Systematic Review. PLoS ONE.

[71] Schutijser B.C.F.M., Klopotowska J.E., Jongerden I.P., Spreeuwenberg P.M.M., De Bruijne M.C., Wagner C. (2019). Interruptions during intravenous medication administration: A multicentre observational study. J. Adv. Nurs..

[72] Havaei F., MacPhee M., Lee S.E. (2019). The effect of violence prevention strategies on perceptions of workplace safety: A study of medical-surgical and mental health nurses. J. Adv. Nurs..

[73] Manapragada A., Bruk-Lee V., Thompson A.H., Heron L.M. (2019). When safety climate is not enough: Examining the moderating effects of psychosocial hazards on nurse safety performance. J. Adv. Nurs..

[74] Kim J., Bates D.W. (2013). Medication administration errors by nurses: Adherence to guidelines. J. Clin. Nurs..

[75] Aveling E.-L., Parker M., Dixon-Woods M. (2016). What is the role of individual accountability in patient safety? A multi-site ethnographic study. Sociol. Health Illn..

[76] DiCuccio M.H. (2015). The Relationship Between Patient Safety Culture and Patient Outcomes: A Systematic Review. J. Patient Saf..

[77] Gurková E., Zeleníková R., Friganovic A., Uchmanowicz I., Jarošová D., Papastavrou E., Žiaková K. (2019). Hospital safety climate from nurses’ perspective in four European countries. Int. Nurs. Rev..

[78] Hessels A.J., Genovese-Schek V., Agarwal M., Wurmser T., Larson E.L. (2016). Relationship between patient safety climate and adherence to standard precautions. Am. J. Infect. Control.

[79] Doronina O., Jones D., Martello M., Biron A., Lavoie-Tremblay M. (2017). A Systematic Review on the Effectiveness of Interventions to Improve Hand Hygiene Compliance of Nurses in the Hospital Setting. J. Nurs. Scholarsh..

[80] Martos-Cabrera M.B., Mota-Romero E., Martos-García R., Gómez-Urquiza J.L., Suleiman-Martos N., Albendín-García L., Cañadas-De la Fuente G.A. (2019). Hand Hygiene Teaching Strategies among Nursing Staff: A Systematic Review. Int. J. Environ. Res. Public Health.

[81] Shimoni Z., Kama N., Mamet Y., Glick J., Dusseldorp N., Froom P. (2009). Empowering surgical nurses improves compliance rates for antibiotic prophylaxis after caesarean birth. J. Adv. Nurs..

[82] Leotsakos A., Zheng H., Croteau R., Loeb J.M., Sherman H., Hoffman C., Morganstein L., O’Leary D., Bruneau C., Lee P. (2014). Standardization in patient safety: The WHO High 5s project. Int. J. Qual. Health Care.

